# Characterization of *Vittatidera zeaphila* (Nematoda: Heteroderidae) from Indiana with molecular phylogenetic analysis of the genus

**DOI:** 10.21307/jofnem-2020-024

**Published:** 2020-03-30

**Authors:** Andrea M. Skantar, Zafar A. Handoo, Mihail R. Kantor, Lynn K. Carta, Jamal Faghihi, Virginia Ferris

**Affiliations:** 1 Mycology and Nematology Genetic Diversity and Biology Laboratory, USDA, ARS, BARC-West, Bldg. 010A, Rm. 111, Beltsville, MD, 20705; 2 Department of Entomology, Purdue University, 901 West State St. West Lafayette, IN, 47907-2089

**Keywords:** Cyst nematode, 18S rDNA, 28S rDNA, Taxonomy, *Vittatidera zeaphila*

## Abstract

In the summer of 2016, a field of corn (*Zea mays*) in Spencer County, Indiana was observed with heavily stunted plants, and from the affected roots a large number of cysts were recovered. Soil samples were submitted to one of us (JF), who extracted the nematode cysts and sent them to the USDA-ARS, Mycology and Nematology Genetic Diversity and Biology Laboratory (MNGDBL), Beltsville, MD for morphological and molecular identification. Cysts and the recovered second-stage juveniles (J2) that were examined morphologically conformed to the measurements of *Vittatidera zeaphila*, the goose cyst nematode originally described from Tennessee, USA in 2010. The molecular analysis of J2 showed the sample from Spencer County matched exactly with *V. zeaphila* according to ribosomal DNA markers ITS, 28S, and 18S, and with mitochondrial cytochrome oxidase I (COI). The nuclear marker heat shock protein 90 (Hsp90) was also analyzed for the first time from the Indiana population of *V. zeaphila*. Similarities to existing cyst nematode sequences are reported herein. Geographically, although the county is across the Ohio River from Kentucky, the previously reported Hickman County, Kentucky location and Indiana detection are approximately 200 miles apart. To the best of our knowledge, this is the first report of *V. zeaphila* in Indiana.

Globally, cyst nematodes species cause serious damage to a wide variety of economically important crops. The need for information on cyst-forming nematode species has been instrumental in stimulating growth of nematology worldwide. The cyst nematode group currently contains eight genera, with a total of 121 valid species (Handoo and Subbotin, 2018). The general morphology and molecular taxonomy and phylogeny of cyst nematodes have been given in detail in two recent review articles (Baldwin and Handoo, 2018; Subbotin and Skantar, 2018). *Vittatidera zeaphila* (Bernard et al., 2010), the goose grass cyst nematode, was first described from Obion County, Tennessee, USA, parasitizing corn and goosegrass. Later, host range studies and potential sources of resistance to *V. zeaphila* were published by Donald et al. (2012). In the summer of 2016, a field of corn (*Zea mays*) in Spencer County, Indiana was observed with heavily stunted plants, and from the affected roots a large number of cysts were recovered. Soil samples were submitted to one of us (JF), who extracted the nematode cysts and sent them to the USDA-ARS, Mycology and Nematology Genetic Diversity and Biology Laboratory (MNGDBL), Beltsville, MD for morphological and molecular identification. Cysts and second-stage juveniles (J2) conformed morphologically and morphometrically to *V. zeaphila.* We report here the first occurrence of this species in Indiana, thus representing the third state after Tennessee, and Kentucky in the United States.

## Materials and methods

### Various stages

Cysts, white females, and J2 were obtained from soil and roots associated with corn plants from Spencer County, Indiana. Juveniles were separated from soil by sieving and Baermann funnel extraction or were collected from cysts removed from fresh roots and kept in water in watch glasses. Juveniles were fixed in 3% formaldehyde and processed to glycerin with a formalin glycerin method (Hooper, 1970; Golden, 1990). Females and some cysts were removed from roots after fixation for 12 hr in 3% formaldehyde solution.

Photomicrographs of cyst vulval cones, and J2 were made with an automatic 35-mm camera attached to a compound microscope having an interference contrast system. Roots and whole cysts were photographed under a dissecting microscope Nikon SMZ18, and light microscopic images of fixed nematodes were taken on a Nikon Eclipse Ni compound microscope using a Nikon DS-Ri2 camera. Measurements were made with an ocular micrometer on a Leica WILD MPS48 Leitz DMRB compound microscope. All measurements are in micrometers unless otherwise stated.

### Nematode DNA preparation

The molecular identification was performed using DNA extracted from single nematodes as template in PCR reactions. Single juveniles were mechanically disrupted with sharp forceps tips in 20 μl nematode extraction buffer (500 mM KCl, 100 mM Tris-Cl (pH8.3), 15 mM MgCl_2_, 10 mM dithiothreitol (DTT), 4.5% Tween 20 and 0.1% gelatin) (Thomas et al., 1997) and stored at −80°C until needed. To prepare DNA extracts, frozen nematodes were thawed, 1 μl proteinase K (from 2 mg/ml stock solution) was added, and the tubes were incubated at 60°C for 60 min, followed by 95°C for 15 min to deactivate the proteinase K. Two or five microliters of extract were used for each PCR reaction.

### PCR amplification and cloning

ITS: Amplification of the internal transcribed spacer region ITS1&2 rDNA contained 0.2 μM each primer, TW81 (Joyce et al., 1994) and AB28 (Howlett et al., 1992), 1.5 mM MgCl_2_, 0.2 mM dNTPs, 1U Platinum Taq DNA polymerase (Invitrogen, Carlsbad, CA), 3 μl nematode DNA extract, and supplied enzyme reaction buffer in a total volume of 25 μl. Cycling included one step of 95°C for 2 min, followed by 35 cycles of 95°C for 30 sec, 55°C for 30 sec, and 72°C for 90 sec, finished with one cycle at 72°C for 5 min (Skantar et al., 2007).

28S: Amplification of the 28S large ribosomal subunit (LSU) D2-D3 expansion segment included the primers D2A [5′-ACAAGTACCGTGAGGGAAAGTT-3′] and D3B [5′-TCGGAAGGAACCAGCTACTA-3′] and were amplified as previously described (De Ley et al., 2005; Ye et al., 2007).

18S: The 18S (small subunit: SSU) sequence was amplified with primers in one fragment with forward primer 18S-CL-F3: [5′-CTTGTCTCAAAGATTAAGCCATGCAT-3′] (Carta and Li, 2018) and reverse primer 1912R according to Holterman et al. (2006). Reactions contained 2 μl nematode DNA extract, 0.5 μl 10 μM primers, 0.5 μl 10 mM dNTP, 1U Platinum Taq DNA polymerase (Invitrogen, Carlsbad, CA), 0.75 μl 50 mM MgCl_2_, and 2.5 μl PCR buffer in a total volume of 25 μl. PCR cycling conditions were 95°C for 3 min, 35X (94°C for 30 sec, 50°C for 40 sec, 72°C for 70 sec), 72°C for 5 min, 4°C until finish.

COI: Mitochondrial cytochrome oxidase I (COI) was amplified with primers Het-coxiF [5′-TAGTTGATCGTAATTTTAATGG-3′] and Het-coxiR [5′-CCTAAAACATAATGAAAATGWGC-3′]. Amplifications were performed in 25 μl reactions with 1x PicoMaxx (Agilent) buffer, 0.2 mM dNTPs, 0.3 μM each primer, 0.125 μl Dream Taq DNA Polymerase (Thermo Fisher), 0.5 μl PicoMaxx Taq, and 3 μl DNA extract. Cycling conditions were as described previously (Subbotin, 2015).

Hsp90: Heat shock protein 90 (Hsp90) fragments were amplified with degenerate primers U288 [5′-GAYACVGGVATYGGNATGACYAA-3′] and L1110 [5′-TCRCARTTVTCCATGATRAAVAC-3′] (Skantar and Carta, 2004). Cycling was performed with 1× PicoMaxx reaction buffer, 0.2 mM dNTPs, 1.5 mM MgCl_2_, 0.3 μM each primer, 1.25 U PicoMaxx Taq, 1 U Platinum Taq, and 3 μl nematode DNA extract. PCR cycling conditions were 94°C 2 min, followed by 45 cycles of [94°C 20 sec, 65°C 5 sec, 60°C 5 sec, 55°C 5 sec, 45°C 5 sec, 68°C 3 min], ending with 1 cycle of 68°C for 15 min.

PCR products were analyzed by electrophoresis on 2% agarose with 1X SB (sodium borate-EDTA) buffer. Gels were stained with ethidium bromide and visualized using the U:Genius gel documentation system (Syngene, Frederick, MD). Hsp90 fragments were cloned using the Strataclone PCR Cloning Kit (Agilent, Santa Clara, CA) according to manufacturer’s instructions. Plasmid clone DNA was prepared with the QiaPrep Spin Miniprep Kit (Qiagen, Valencia, CA) and digested with Eco RI to verify the presence of insert. Sequencing was performed by Genewiz, Inc. Direct sequencing of PCR amplicons was used to obtain the 28S sequences (assigned GenBank accession numbers MK121965-MK121968), the ITS 1&2 rDNA sequence (MK121952), the 18S rDNA sequence (MK182465), and COI sequences (MK253554-MK253558). Accession numbers were assigned for new sequences from cloned Hsp90 of *V. zeaphila* (MK580824-MK580829), *Heterodera avenae* (MH484608, MH484609, and MH484611), and the outgroup *Helicotylenchus digonicus* (MK580830).

### Phylogenetic inference and tree visualization

Raw sequence reads were processed in Sequencher 5.4.6 (Genecodes, Inc., Ann Arbor, MI). Multiple DNA sequence alignments were created using Geneious Prime 2019.0.3 (www.geneious.com) with built-in parameters or the MAFFT plug-in, with auto-selection of best algorithm depending on data. Alignments of Hsp90 intron regions were adjusted further using Geneious Alignment with free end gaps and identity (1.0/0.0) cost matrix and inspected visually to ensure preservation of exon boundaries and reading frames. Phylogenetic analysis using Bayesian inference (Huelsenbeck and Ronquist, 2001) was performed via the CIPRES Gateway (Miller et al., 2010), except where noted otherwise. The model of nucleotide evolution was determined by jModelTest 2.1.3 (Darriba et al., 2012), with the best-fit model GTR+I+G selected for Hsp90. The parameters for base frequency, proportion of invariable sites, and gamma distribution shape, and substitution rates according to Akaike Information Criteria (AIC) generated in a custom command block implemented in MB. Bayesian analysis was run with four chains for 2 × 10^6^ generations, with Markov chains sampled at intervals of 500 generations. Two runs were performed for each analysis. After burn-in samples were discarded and convergence evaluated, the remaining samples were retained for further analysis. Topologies were used to generate 50% majority rule consensus trees with posterior probabilities given on appropriate clades. Trees were visualized in Geneious. Alternative alignments of Hsp90 genomic sequences or coding regions were made using Clustal Omega (Sievers et al., 2011) and MUSCLE (Edgar, 2004) plug-ins for Geneious, and corresponding trees were made in MB or IQ-TREE (Nguyen et al., 2015) to investigate the effect of removing introns or partitioning the data on tree topology and support values.

COI alignments were likewise made within Geneious, using *Atalodera carolynae* as the outgroup. Phylogenetic reconstruction based on COI alignments were performed in IQ-TREE, within which ModelFinder (Kalyaanamoorthy et al., 2017) determined the best-fit model to be K3Pu + F + I + G4. The consensus tree was constructed from 1,000 bootstrap trees using UFBoot2 (Hoang et al., 2018) with bootstrap values indicated on appropriate branches. Alternative MB trees were made for comparison, including runs where rate parameters were independently specified for codon position.

## Results and discussion

### Description

#### Morphology and measurements

**Figure 1:**
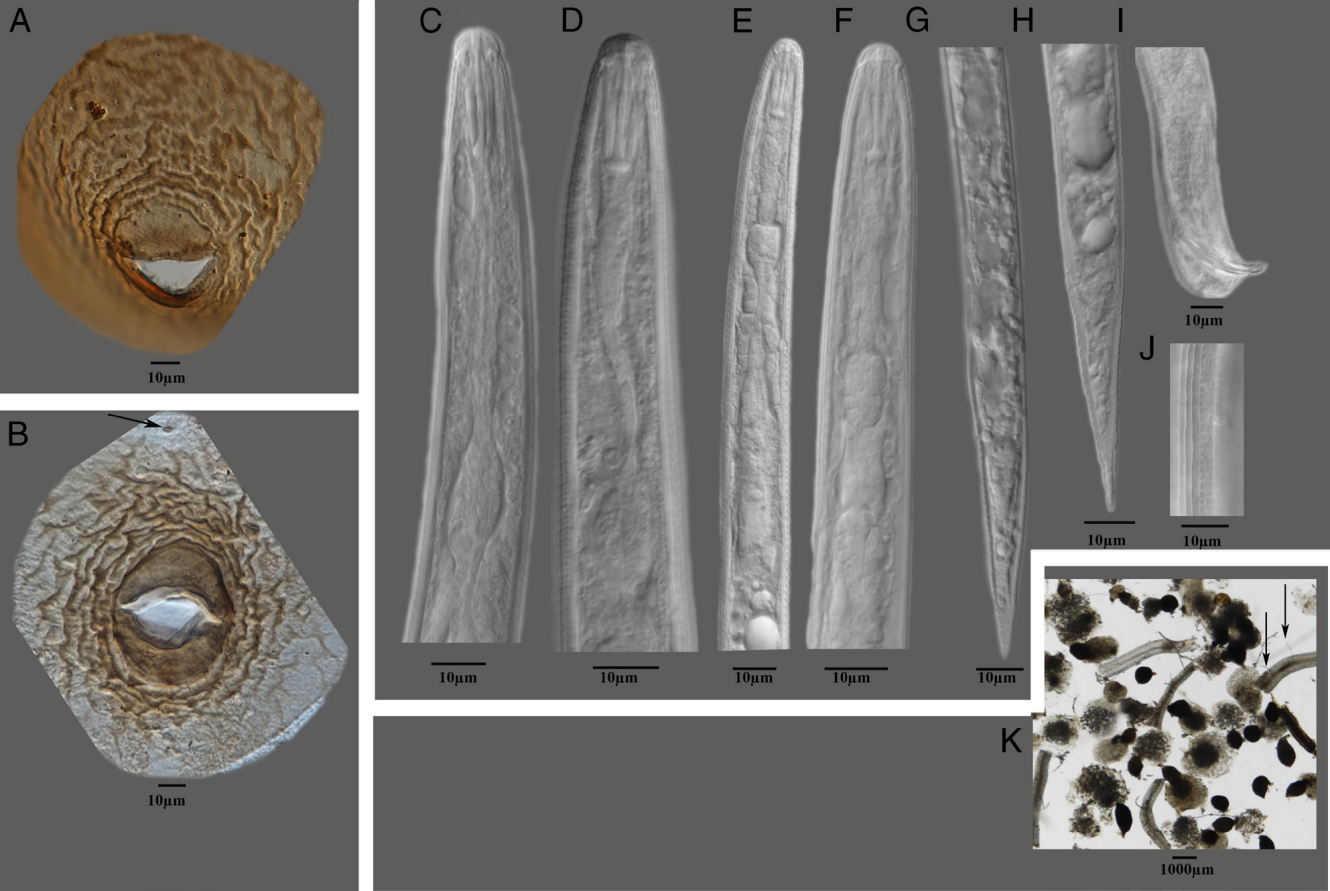
Photomicrographs of *Vittatidera zeaphila* population on Corn (*Zea mays*) from Spencer County, Indiana. (A, B): light micrograph of vulval cones of *V. zeaphila* showing fenestra area with arrow showing the anal area, (C, D): males anterior ends showing head and esophageal regions, (E, F): second-stage juveniles anterior ends showing head and esophageal regions, (G, H): second-stage juveniles posterior ends showing tail and tail terminus, (I): male posterior end showing the spicule, (J): lateral field with four lines at mid-body, (K): cysts with attached egg masses on corn roots with arrows showing second-stage juveniles feeding on corn roots.

Photomicrographs of *V. zeaphila* from Spencer County, Indiana are shown in Figure 1. Measurements of second-stage juveniles (*n* = 10) included length of body (range = 332-385 μm, mean = 351 μm). Lip region rounded, slightly set off with three to four annules, stylet short, well developed (15.0-16.0 μm, 15.8 μm) with rounded knobs, lateral field with four distinct lines, tail elongate conoid with narrowly rounded terminus (37.0-48.0 μm, 42.1 μm), and hyaline tail terminus (11-15 μm, 13.1 μm). Shapes of the tail, tail terminus, and stylet knobs were consistent with *V. zeaphila*. The cysts were lemon-shaped, dark to reddish light brown in color; vulval cone was not protuberant, with large egg masses (Fig. 1K); mature cysts had vulval cone circumfenestrate, underbridge, bullae absent. Morphometrics of cysts (*n* = 5) included body length (L) including neck (520-866 μm, 696 μm); body width (W) (320-495 μm, 399.8 μm; L/W (1.4-2.1, 1.7 μm); neck length (60-100 μm, 74.0 μm) and width (45-55 μm, 50.0 μm); fenestra length (52-65 μm, 58.4 μm) and width (32-40 μm, 37.4 μm). The cysts had light to wavy line type of cyst wall cuticular pattern (Fig. 1A,B); anus opening was prominent; punctations often present in terminal area of cyst; morphometrics of cysts were also consistent with *V. zeaphila*. Lateral field distinct in both females and cyst stages, arched, represented by short transvers lines between neck and cone. In female perineal region phasmid present.

Living nematode juveniles (J2) collected from the cysts were examined morphologically and molecularly for species identification. Observations of morphological characters critical for identification (Fig. 1C-J) indicated that the specimens agreed with the previous *Vittatidera zeaphila* description by Bernard et al. (2010), except for minor morphometric difference in the head end to excretory pore distance which is closer to 76.7 (70-80 μm) vs 148 (142-161 μm) in the original description, and which according to scale bar measurements should be 74 (71-80.5).

#### Molecular characterization

Three sequences of ITS 1&2 rDNA obtained from separate J2 were assembled into a 936 bp alignment of identical sequences that overlapped with a 540 bp region from JF741961 previously described from the TN population of *V. zeaphila* (Bernard et al., 2010). Except for a few ambiguous positions in the latter sequence (possible sequencing artifacts), the ITS sequences from both populations were identical. 28S rDNA sequences obtained from four J2 were assembled into a 762 bp alignment along with JF741960 previously described from the TN population of *V. zeaphila*, differing at 0 to 2 bp from each other and from the TN sequence. The 18S rDNA sequence was obtained from amplification of the gene from a single J2. A 985 bp alignment with the TN *V. zeaphila* sequence (JF741962) showed that the 18S sequences were identical. For Hsp90, six sequences were obtained from cloning the amplicons from a single J2. Sequence lengths were 1412 bp with four introns. Excluding the degenerate primer regions at each end, the sequences varied from 4 to 11 bp among the clones, mostly within introns or at third codon positions. No Hsp90 sequence was available from the TN population for comparison.

**Figure 2:**
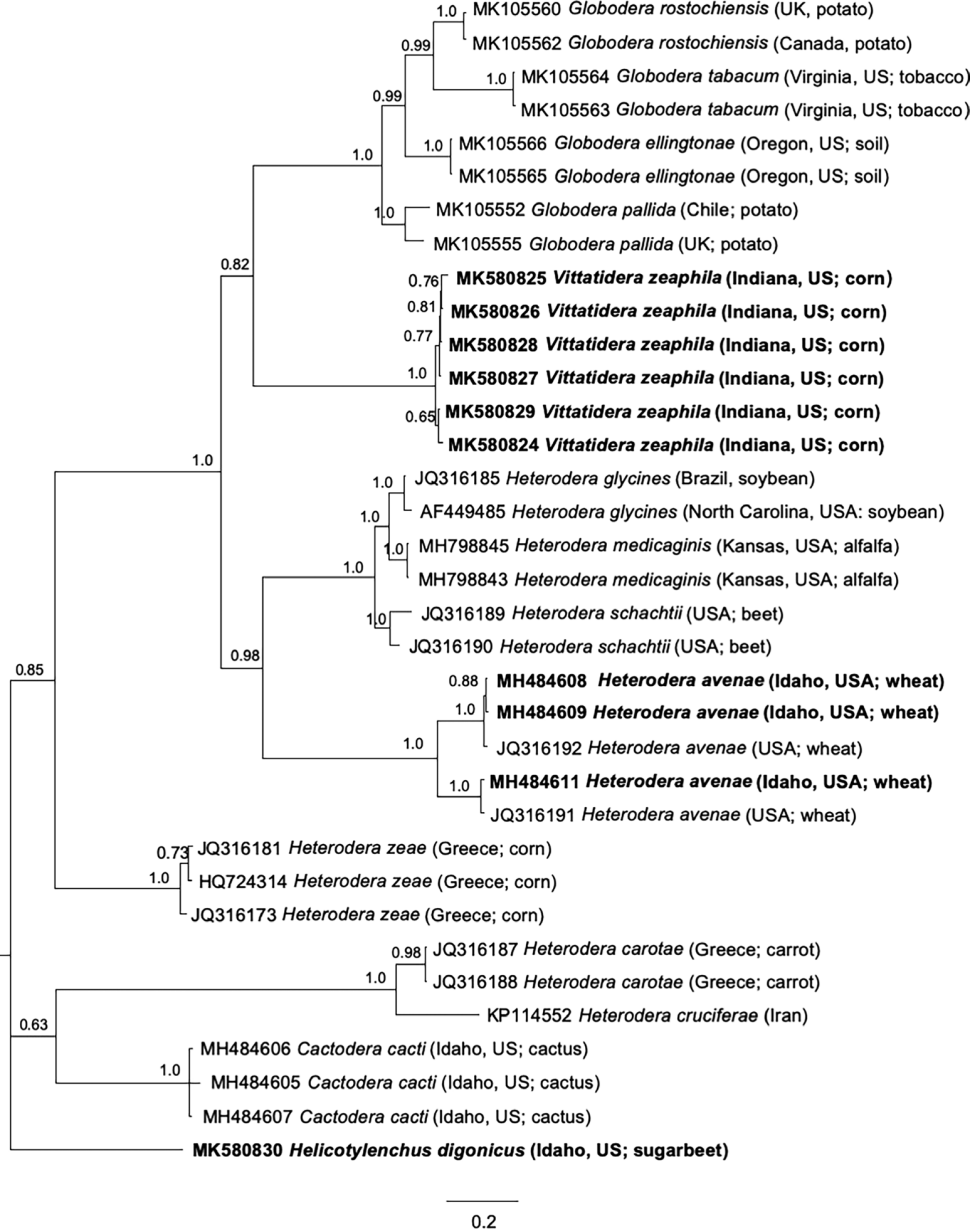
Phylogenetic relationships of *Vittatidera zeaphila* and other selected cyst nematodes, as inferred from a 1887 bp alignment of partial Hsp90 genomic DNA sequences, with *Helicotylenchus digonicus* as the outgroup. A 50% majority rule consensus tree obtained from Bayesian analysis was generated using the GTR + I + G model of nucleotide substitution. Branch support values above 50% are shown on appropriate branches. New sequences are highlighted in bold.

Phylogenetic relationships among heteroderid nematodes were inferred from analysis of 35 partial Hsp90 sequences from 14 species, in a genomic alignment of 1887 bp (Fig. 2). *Vittatidera zeaphila* sequences had two introns (273 bp and 200 bp) that were significantly longer than the corresponding introns from other cyst nematodes in the alignment. *Vittatidera zeaphila* formed a sister group to the *Globodera* clade, although support was moderate (0.82). Consistent with previously published 18S, 28S, and ITS trees (Bernard et al., 2010), the Hsp90 tree placed *V. zeaphila* genetically distant from the other cyst nematode of corn, *Heterodera zeae* (Fig. 2). *Cactodera cacti* grouped apart from the other Punctoderinae, appearing in a clade that included the Goettingiana group, but with weak support (0.63). To assess the effect of additional taxa on topologies, another tree was constructed from a shorter, 594 bp genomic alignment of 50 sequences; this tree resulted in weaker support for the position of *V. zeaphila* relative to the *Globodera* clade and it did not further resolve the other clades (not shown). This is most likely due to the shorter alignment containing fewer informative characters. Moreover, a third alignment that included only coding regions with introns removed contained essentially the same topology and branch support as seen in Figure 2, even when codon positions were modeled independently (not shown). In previous studies with trees inferred from ITS rDNA, *V. zeaphila* was not resolved in relation to the rest of the Punctoderinae (Subbotin and Skantar, 2018), so the long Hsp90 fragment analyzed here performed as good or better, considering the relative lack of available sequences for analysis compared to ITS.

**Figure 3:**
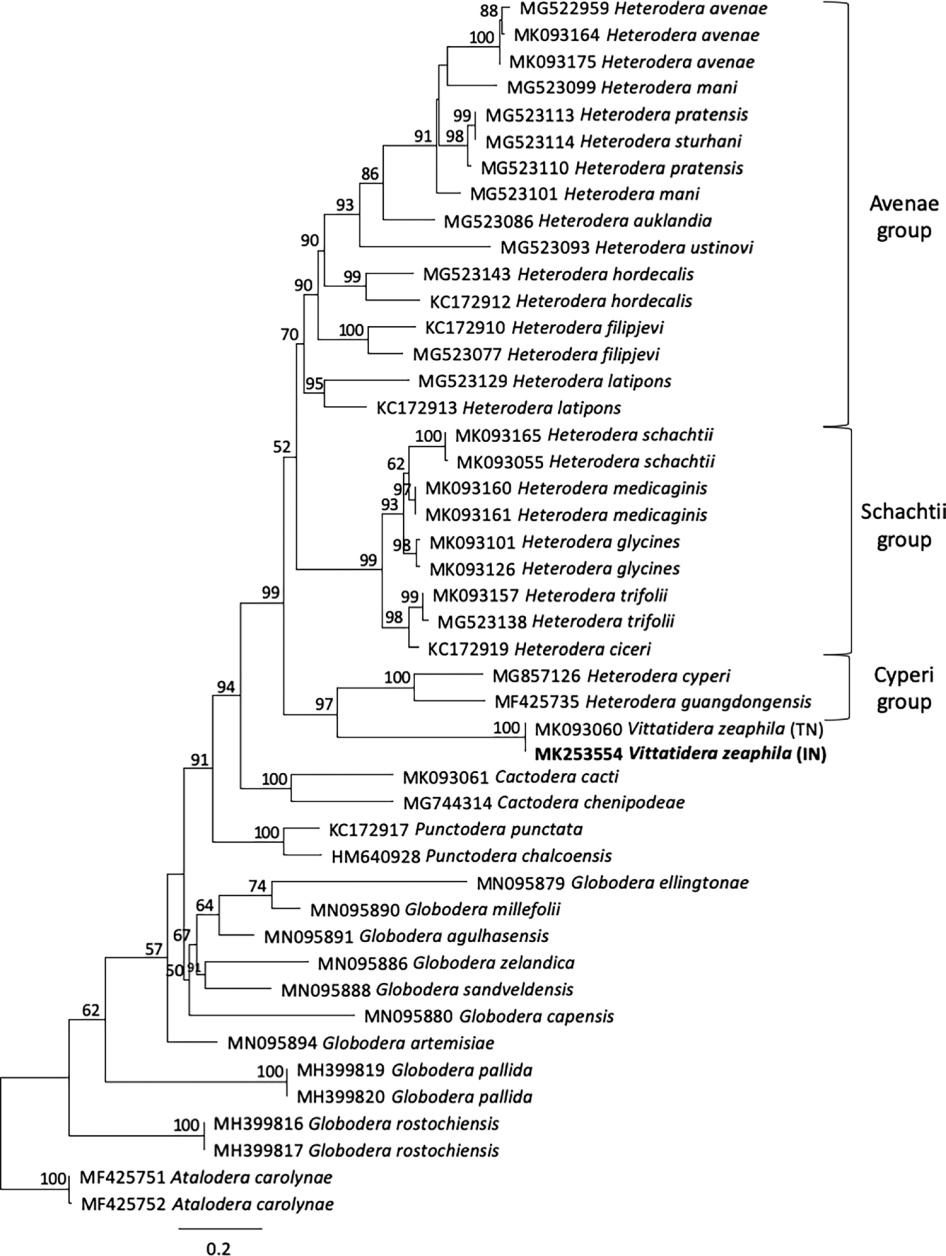
Phylogenetic relationships of *Vittatidera zeaphila* and other select cyst nematodes, as inferred from a 373 bp alignment representing 33 taxa, with *Atalodera carolynae* as the outgroup. The tree was constructed in IQ-TREE using model K3Pu + F + I + G4; the consensus tree was constructed from 1,000 bootstrap trees using UFBoot2. Bootstrap values are included on appropriate branches. New sequences are highlighted in bold.

COI sequences obtained from five J2 were assembled into a 456 bp alignment. All sequences were identical to each other and were 100% match to *V. zeaphila* from Tennessee (MK093060). BlastN showed that the next closest sequence matches had maximum similarity of only 82% to *H. glycines* and other *Heterodera* species. In total, 46 selected cyst nematode COI fragments were assembled in a trimmed 373 bp alignment representing 33 taxa, with the resulting IQ-TREE shown in Figure 3. In this analysis, *V. zeaphila* appeared as a sister taxon to the Cyperi group of Heteroderinae, with 97% support. Taxa within the Avenae and Schachtii groups were also strongly supported and agreed with trees based upon ITS rDNA (Subbotin and Skantar, 2018). A number of additional analyses using MB parameters under the GTR+I+G model adjusted to include more or fewer taxa left *H. filipjevi* and *H. latipons* in unresolved positions relative to the rest of the Avenae group, which disagreed with previous phylogenies (Subbotin and Skantar, 2018). Relationships among *Globodera* spp. were not conclusively resolved by COI, possibly due to gene duplications and variability within the multipartite mitochondrial genomes in those species (Armstrong et al., 2000; Phillips et al., 2016). The relative position of *V. zeaphila* relative to *Cactodera* spp. and *Globodera* spp. was best supported in the tree shown in Figure 3. In a prior study focused primarily on the relationship of *H. medicaginis* relative to populations of *H. glycines*, a COI tree placed *V. zeaphila* in a weakly supported clade with *Meloidodera* spp., but its relationship relative to *Punctodera* spp. and *Cactodera* spp. was not well resolved (Powers et al., 2019). An 18S tree from another study placed *V. zeaphila* in a clade with a single *C. betulae* sequence, but could not resolve its relationship to the clade containing *Globodera* spp. and *Heterodera* spp. (DeLuca et al., 2013). The 28S tree from Bernard et al. (2010) contained a wider range of heteroderid genera for comparison, with *V. zeaphila* as a sister taxon to *Heterodera*, *Cactodera*, *Punctodera*, *Globodera*, and *Dolichodera.* The relationship of *V. zeaphila* to the other circumfenestrate nematodes remains fluid and will be strengthened as additional sequences become available.

Although evidence of damage to host plants from *V. zeaphila* was previously established (Donald et al., 2012), there remains an urgent need to investigate the economic impact of this nematode on corn in order to develop sustainable management strategies.
